# Vinegar inhibits the formation of oral biofilm in situ

**DOI:** 10.1186/s12903-020-01153-z

**Published:** 2020-06-05

**Authors:** Yong Liu, Matthias Hannig

**Affiliations:** Clinic of Operative Dentistry, Periodontology and Preventive Dentistry, Saarland University, 66421 Homburg, Germany

**Keywords:** Vinegar, Biofilm, In situ, Pellicle

## Abstract

**Background:**

Vinegar has been recognized as an effective antimicrobial agent for long. This study intended to elucidate the effect of commercially available vinegar on in situ pellicle formation and existing 24-h biofilms.

**Methods:**

In situ biofilm formation took place on bovine enamel slabs mounted in individual splints and exposed intraorally over 3 min and 24 h, respectively. After 5 s rinsing with vinegar, all samples were analyzed via fluorescence microscopy (FM), scanning electron microscopy (SEM) and transmission electron microscopy (TEM). In addition, salivary samples were collected and analyzed via FM. Samples with water rinsing served as controls.

**Results:**

Vinegar caused destruction of the pellicle. Compared to the control group, vinegar rinsing reduced the outer globular layer of the pellicle (*p* < 0.001), and resulted in formation of subsurface pellicle. Also, vinegar rinsing could reduce bacterial viability and disrupt the 24-h biofilm. Total bacteria amount of saliva samples decreased remarkably (*p* < 0.001) after vinegar rinsing within 30 min. Reduction of bacterial viability was observed even 120 min after vinegar rinsing in both biofilm and saliva sample (*p* < 0.001).

**Conclusion:**

This in situ study reveals that rinsing with vinegar for only 5 s alters the pellicle layer resulting in subsurface pellicle formation. Furthermore, vinegar rinsing will destruct mature (24-h) biofilms, and significantly reduce the viability of planktonic microbes in saliva, thereby decreasing biofilm formation.

## Background

Dental problems have been the major reason of Years Lived with disability throughout the world [1]. Oral biofilm has been accepted to be the main reason of oral diseases, such as caries and periodontitis which are caused by dysbiosis and imbalance of the biofilm’s composition [2]. Therefore, biofilm management takes an essential part in prevention and treatment of oral diseases.

Among many other natural antibacterial agents, vinegar is often used for the prevention and treatment of diseases because of its low pH value. Different studies have reported that ear irrigation with diluted vinegar can be effective in the treatment of ear infections, such as chronic suppurative otitis media [3], granular myringitis [4] and otitis externa [5]. Also, the undiluted vinegar can effectively remove the bacteria from dentures, which will not cause oral mucosal damage even if residual vinegar remains on the denture [6]. The explanation for this might be that vinegar affects cell membrane function, leading to transmembrane proton motility destruction [7, 8], as well as other factors such as inhibition of enzyme activity [9], energy competition and inhibition of bacterial protein expression [10].

Although vinegar has such antibacterial effects, there is few research of vinegar applied to the oral biofilm. The impact of vinegar on the initial microflora adherence to enamel and the formation of biofilm in situ have not yet been systematically analyzed. Thus, the present study aims to investigate the effect of commercially available vinegar on in situ formation of the initial pellicle and on 24-h biofilms, as well as on salivary bacteria.

## Methods

### Subjects

Four healthy volunteers aged 25–35 participated in this study. Visual oral examination was carried out by one experienced dentist, including determination of the physiological salivary flow rate. The subjects showed no signs of caries or periodontal disease. Furthermore, informed consent forms were signed before experiment performance, including customary diet, no smoking and no antibiotics usage within 6 months. The study protocol had been approved by the Medical Ethics Committee of the Medical Association of Saarland, Germany (# 238/03, 2016).

### Preparation of specimens and saliva

Bovine permanent incisors from 2-year-old cattle were extracted and prepared to enamel slabs: 4 × 4 mm for *Bac*Light™ viability assay and scanning electron microscopy (SEM), 2 × 2 mm for transmission electron microscopy (TEM). The prepared tooth samples were stored in 0.1% thymol solution (pharmacy of the Saarland University Hospital, Homburg, Germany) at 4 °C. In order to standardize the samples, the surface of the enamel specimens was ground flat and polished under water-cooling (P600-P4.000, FEPA-P, waterproof silicon carbide paper, Buehler, Düsseldorf, Germany) [11–13]. To remove residues of the polishing process, samples were rinsed with 3% NaOCl solution (Hedinger, Stuttgart, Germany) for 3 min and then washed twice in distilled water. Afterwards, the specimens were disinfected by 70% propanol for 15 min [11, 14], and then stored in distilled water for 24 h at 4 °C.

Saliva was collected between 9 and 12 a.m., thereby 2 ml saliva were taken within 5 min. Following this, each sample was measured by means of pH test strips (Macherey-Nagel, Carl Roth GmbH +Co, Karlsruhe, Germany). Subsequently, samples were centrifuged at 1000 rpm for 10 min, and the supernatant was kept for the second centrifugation (10,000 rpm for 10 min). The supernatant was carefully removed and the pelleted bacteria were stained before observed by fluorescence microscopy (FM).

### Formation of in situ biofilm, application of vinegar

**Fig. 1:**
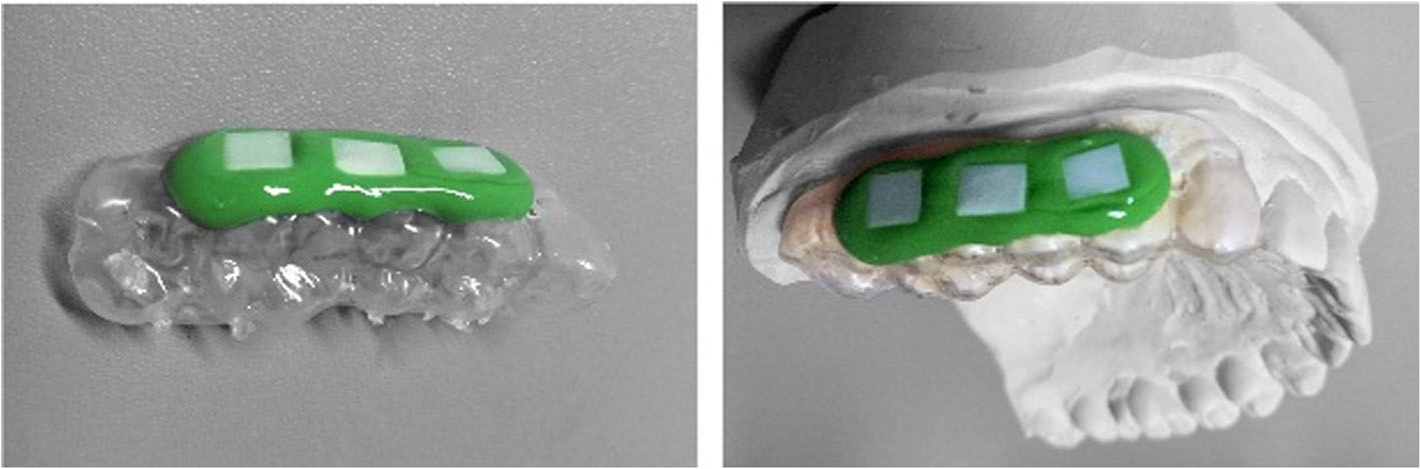
Splint with mounted specimens

For in situ biofilm, individual acrylic upper splints for holding enamel samples were manufactured for the maxillary dentition of all subjects. After disinfection, enamel slabs were mounted to the defined position on the splints by polyvinyl-siloxane impression material (President light-body, Coltene, Altstätten, Switzerland) (Fig. 1).

**Fig. 2:**
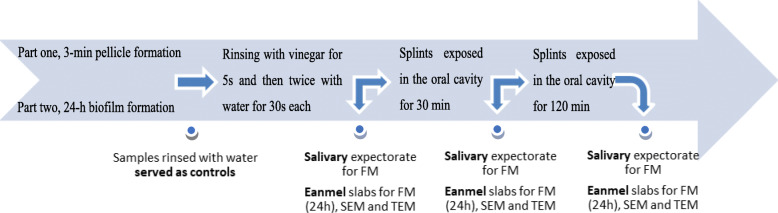
Flowchart of the experiments, in situ formation of 3-min pellicle and 24-h biofilms, and subsequent analyses

The experiment took place at 9 a.m. All the participants were informed to brush their teeth without toothpaste 24 h beforehand and during the experimental period. After the specimens were exposed to the oral cavity for 3 min or 24 h, the volunteers rinsed with 10 ml vinegar (5% distilled vinegar, Heuschen & Schrouff OFT B.V. Thailand) for 5 s and then rinsed twice with 10 ml water for 30 s. Immediately, three enamel slabs were dismounted from the splints and analyzed via FM, SEM and TEM. In the following, the remaining enamel specimens were exposed to the oral cavity for another 30 min or 120 min. Salivary samples were collected when enamel slabs were dismounted. Samples rinsed with water instead of vinegar served as controls. Details of the experimental design are seen in the flow chart (Fig. 2).

### Vital fluorescence microscopy

Enamel slabs covered by 24-h biofilms as well as salivary samples were processed by *Bac*Light™ viability assay (LIVE/DEAD® *Bac*Light™ Bacterial Viability Kit, Art. No. L7012, Invitrogen, Molecular probes, Eugene, Oregon, USA). The staining solution was prepared by 1 μl of SYTO 9 (green) and 1 μl of propidium iodide (PI, red) in 1 ml of 0.9% saline solution. SYTO 9 stain generally labels all bacteria, including those with intact membranes as well as those with damaged membranes. PI binds to double-stranded DNA, and can only penetrate the bacteria if the integrity of the membrane is compromised. PI causes a reduction of the SYTO 9 stain fluorescence in damaged bacteria, when both dyes are present. Therefore, PI-positive (red) staining indicates dead bacteria, while SYTO 9-positive (green) staining indicates live bacteria.

Specimens were stained with 0.1 ml of coloring solution at room temperature in the dark for 15 min. After removal of the residual staining solution, the dried samples were positioned on glass slides covered with mounting oil. The salivary samples were stained by 20 μl of the staining solution at room temperature for 10 min in the dark, and then 1 μl of the mixture was transferred on the slide for analysis.

Samples were evaluated via a fluorescence microscope (Leica DMRB, Leica Mikroskopie & SystemeGmbH, Wetzlar, Germany). Nine images were taken from each sample and quantified via Image J (Image J-ij133- jdk15, National Institute of Mental Health).

### SEM

The enamel slabs were fixed in 2.5% glutaraldehyde solution at 4 °C for 1 h and then washed 5 times with phosphate buffered saline for 10 min each. Subsequently, the samples were dehydrated in a series of 50–100% ethanol solutions. Large particulate matter (e.g. bacteria, protein) on pellicle samples were analyzed regarding the gray value by Image J.

### TEM

Pellicle/biofilm samples were analyzed by TEM in order to visualize the influence of vinegar on the ultrastructure of pellicle and biofilm. After the enamel slabs were removed from the splints, they were fixed in 2.5% glutaraldehyde solution at 4 °C for 1 h and then washed 5 times in cacodylate buffer. Afterwards, the samples were placed in 2% osmium tetroxide for 1 h and then dehydrated in ethanol with a rising gradient concentration. Specimens were embedded in Araldite CY212 (Agar Scientific, Stansted, United Kingdom). After decalcification of the enamel in 1 M HCl, specimens were re-embedded in Araldite. Ultrathin-sections (about 50–80 nm) were cut in an ultramicrotome (Ultracut E, Reichert, Bensheim, Germany) equipped with a diamond knife (Microstar 45°, Plano GmbH, Wetzlar, Germany). Ultrathin sections were mounted on Pioloform-coated coppers grids and contrasted with uranyl acetate and lead citrate. The specimens were investigated in a TECNAI 12 Biotwin TEM (FEI, Eindhoven, Netherlands) under magnifications of 6800 up to 180,000.

### pH value

The salivary samples and vinegar were assessed with pH test paper (Macherey-Nagel, Carl Roth GmbH +Co, Karlsruhe, Germany).

### Statistics

The data were evaluated by the Mann- Whitney U test and Kruskal- Wallis test using SPSS 18 software package (SPSS Inc., Chicago, IL., USA).

## Results

### 3-min pellicle

**Fig. 3:**
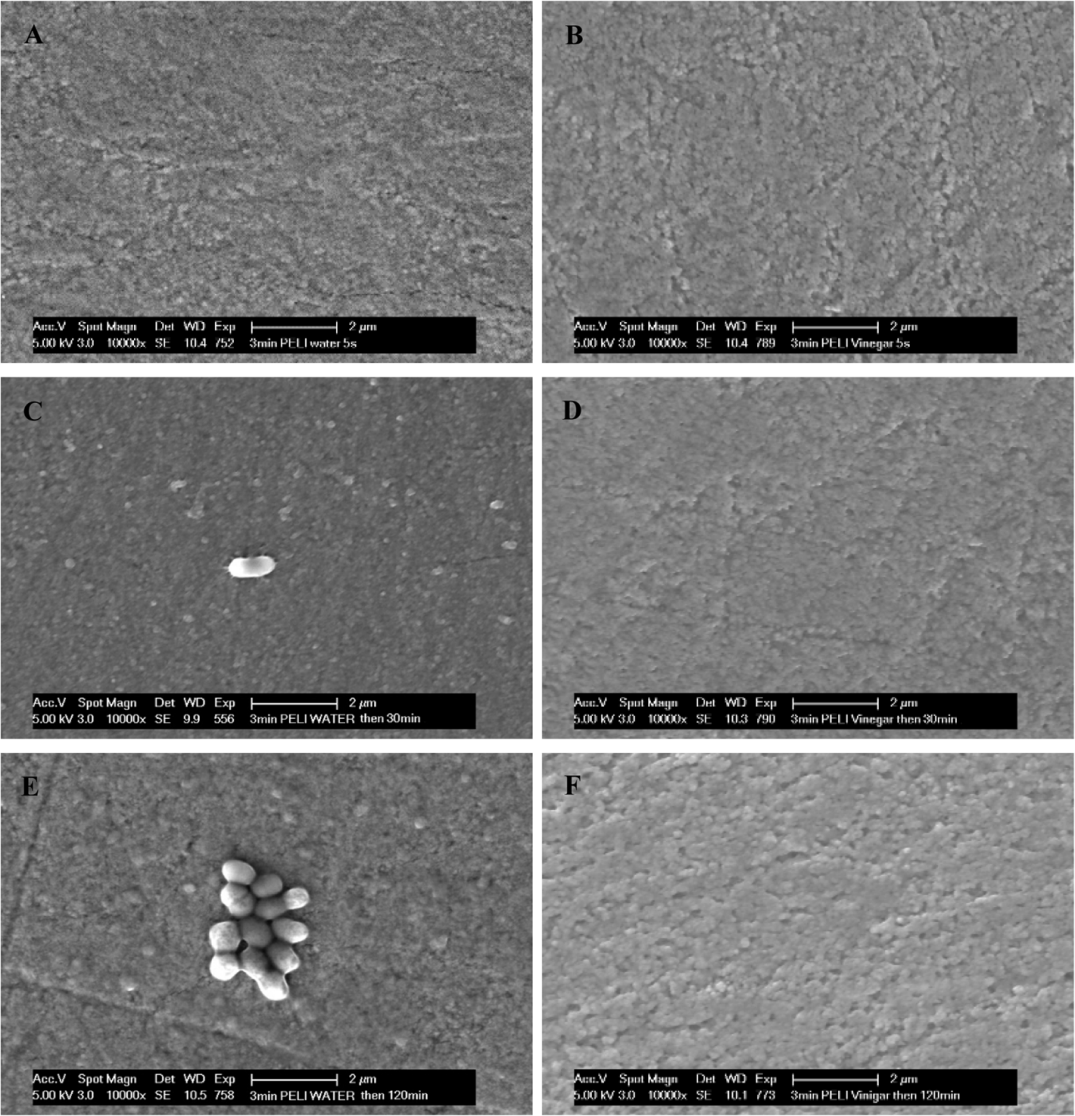
SEM micrographs: an overview of 3 min pellicle immediately, 30 min and 120 min after water rinsing or application of vinegar. **a**, **c** and **e** Control group, globular formation of pellicle is clearly visible; **b**, **d** and **f** Vinegar group, very smooth and clean enamel surface; **a** and **b** immediately after rinsing; **c** and **d** 30 min after rinsing; **e** and **f** 120 min after rinsing. Original magnification: 10,000-fold

SEM images presented the top-view of the enamel surface with significant differences between the vinegar group and the control group. After water rinsing, the numbers of globular pellicle particles increased over time indicating the formation of the outer pellicle layer. In comparison, after vinegar rinsing, there were almost no globular particles to be found up to 120 min suggesting that pellicle formation was strongly inhibited at least for 2 hours (Fig. 3).

**Fig. 4:**
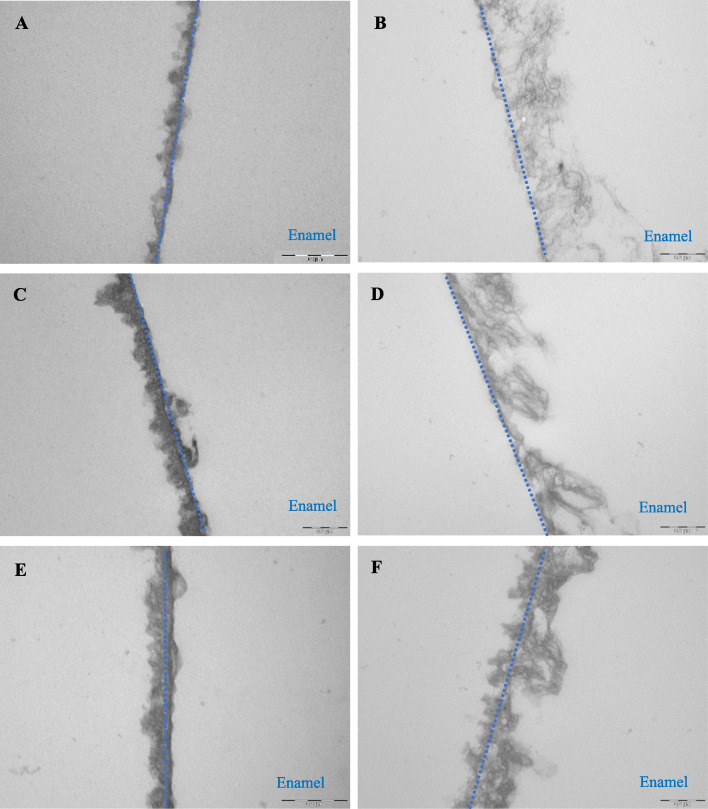
TEM micrographs: gallery of representative pellicle layers after water rinsing and rinsing with vinegar. The blue lines indicated the electron dense layer. “Enamel” indicates the former enamel substrate dissolved during processing of the specimens for TEM analysis. **a**, **c** and **e** Control group, the outer globular layer increased over time, also, the electron-dense basal layer was observed quite thin; **b**, **d** and **f** Vinegar group, the granular structured layer was removed and the subsurface was detected with increasing electron density after 120 min; **a** and **b** immediately after rinsing; **c** and **d** 30 min after rinsing; **e** and **f** 120 min after rinsing. Original magnification: 23,000-fold

TEM micrographs provided ultrastructural details on the internal structures of the 3-min pellicle. The control pellicle layer manifested itself as an electron-dense basal pellicle layer and an outer less dense globular layer. After water rinsing, the thickness of the globular layer was increased over time. In contrast, after vinegar rinsing, this globular layer was completely removed and a thin discontinuous layer can be seen only after 120 min of oral exposure. More interestingly, after vinegar rinsing, a network-like subsurface layer was detected (Fig. 4).

**Fig. 5:**
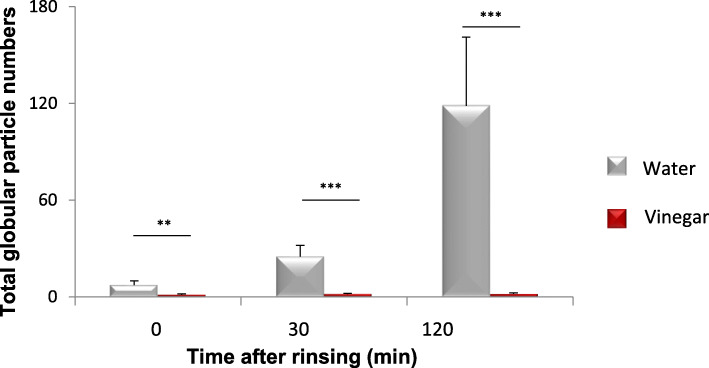
Total globular particle numbers of the 3-min pellicle detected by SEM were counted. The enamel surface kept quite clean after rinsed with vinegar within 120 min over time. After rinsing with water, there were significant differences compared to the vinegar group immediately (*p* < 0.01), in 30 min (*p* < 0.001) and in 120 min (*p* < 0.001) after rinsing. ** *p* < 0.01, ***p < 0.001

The numbers of total globular particles in SEM images on enamel surface were quantified. After water rinsing, the number of particles increased after 30 min (*p* < 0.001) or 120 min (*p* < 0.001), whereas after vinegar rinsing, particle numbers were very close to zero up to 120 min (Fig. 5).

### 24-h biofilm

**Fig. 6:**
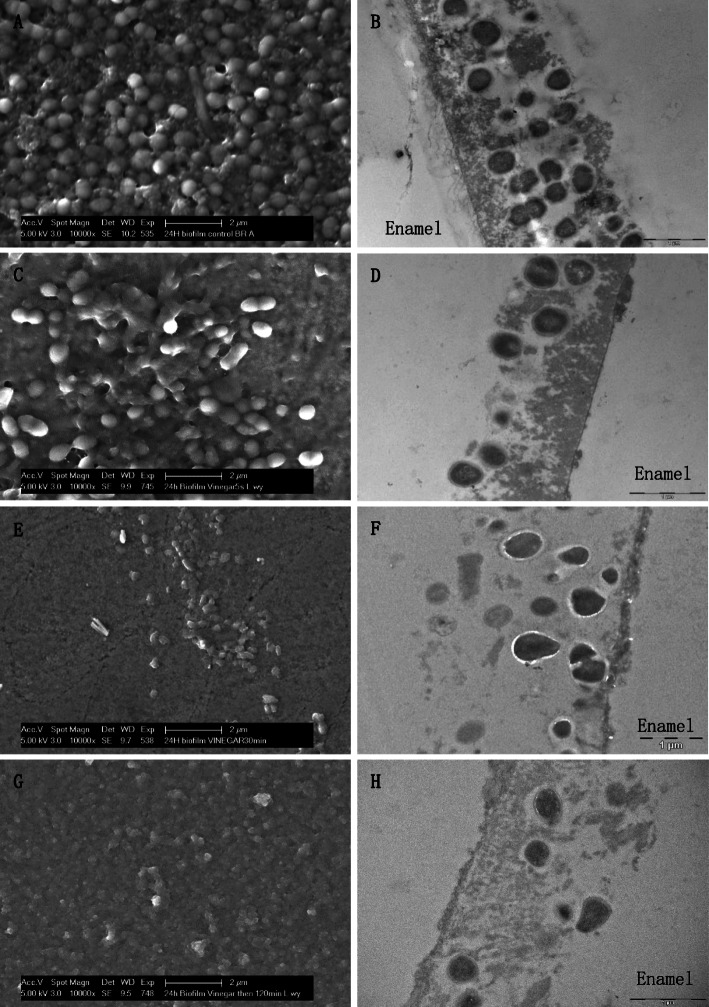
An overview of the 24-h biofilm after application of vinegar. “Enamel” indicates the former enamel substrate dissolved during processing of the specimens for TEM analysis. **a**, **c**, **e** and **g** SEM micrographs. After application of vinegar, the tight connection among bacteria was destroyed and bacteria shape was broken. Original magnification: 10,000-fold. **b**, **d**, **f** and **h** TEM micrographs. Original magnification: 23,000-fold. **a** and **b** Control group; **c** and **d** immediately after rinsed with vinegar; **e** and **f** 30 min after rinsed with vinegar; **g** and **h** 120 min after rinsed with vinegar

**Fig. 7:**
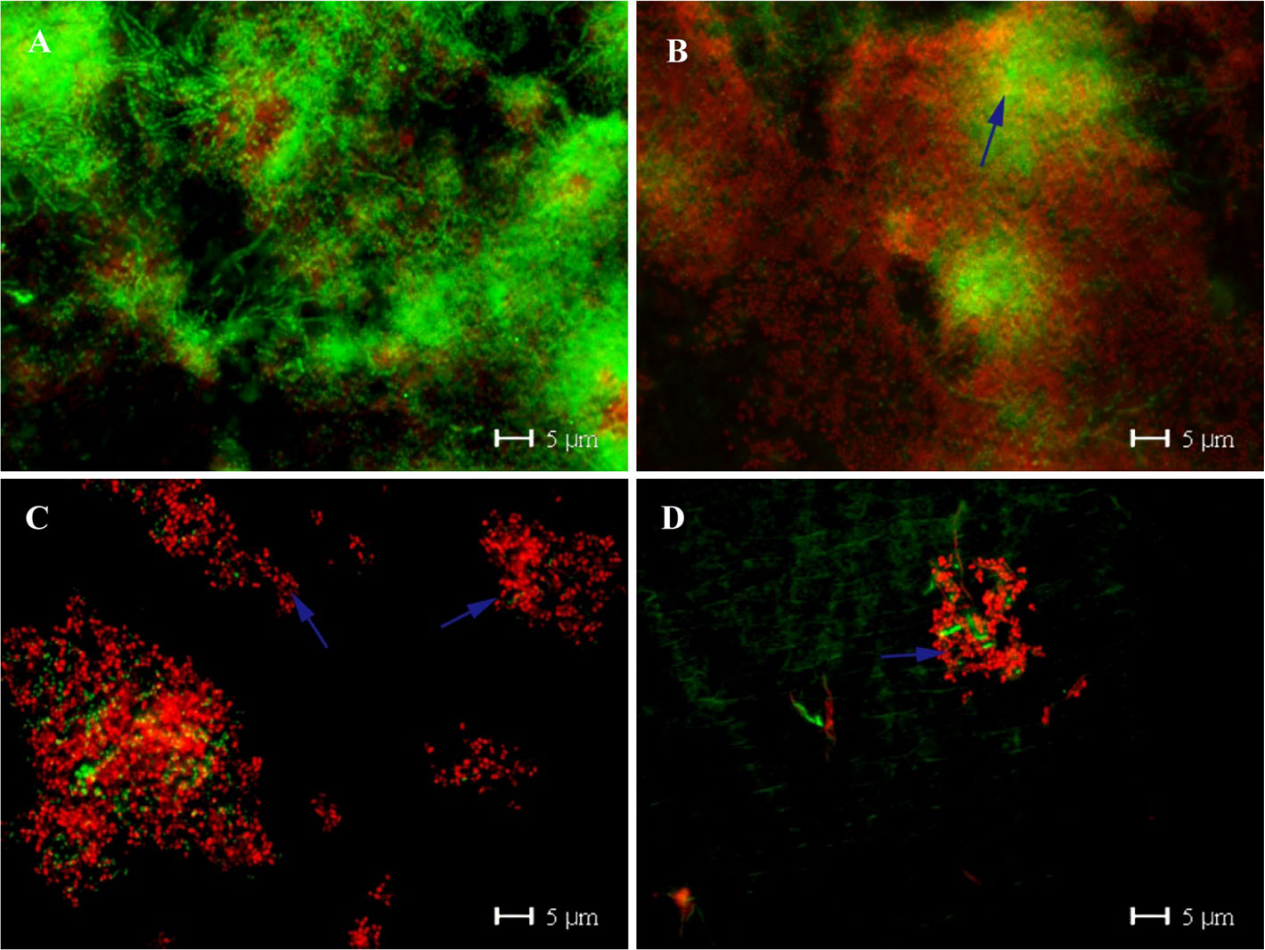
*Bac*Light™ viability assay images of the 24-h biofilm (Control **a**) immediately (**b**), 30 min (**c**) and 120 min (**d**) after rinsing with vinegar. After rinsed with vinegar, bacteria died from the edge of the colony firstly and the large bacterial colony was separated into small ‘islands’. Original magnification: 1000-fold

**Fig. 8:**
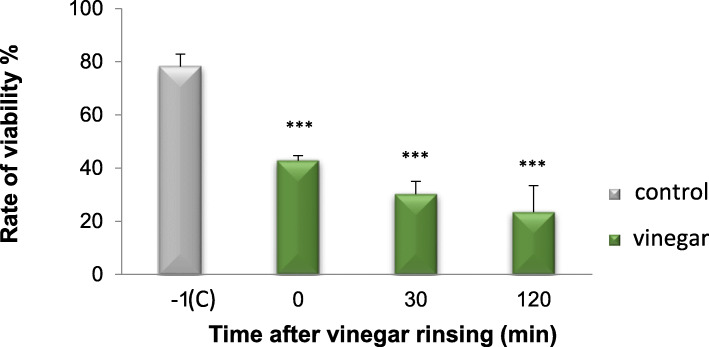
*Bac*Light™ viability assay for determination of bacterial viability of the 24-h biofilm before and after vinegar rinsing at different times. A significant reduction of bacteria viability as compared to the control was shown after vinegar rinsing for 5 s (*p* < 0.001), after 30 min (*p* < 0.001) and after 120 min (*p* < 0.001). ***p < 0.001

The typical pattern of the 24-h control biofilm covering the entire enamel surface, with different densities of bacterial colonies consisting of coccoids and some rod-shape bacteria was detected in SEM micrographs. Immediately after vinegar rinsing, the number of bacteria was reduced, but only mildly. Interestingly, 30 min after vinegar rinsing, the biofilm was almost wiped out, even the biofilm matrix was in part disrupted. It almost remained like that until 120 min. TEM images provided a cross-section view of the biofilm ultrastructure. Comparing to the multi-layer of the control biofilm, bacteria were reduced after vinegar rinsing (Fig. 6). Furthermore, bacterial viability was detected by *Bac*Light™ viability assay. Live bacteria were stained in green and dead in red. After vinegar rinsing, most of the bacteria were already dead, although they still attached to the surface (Fig. 7). The quantification shows that the bacterial viability was considerably reduced. Impressively, even 120 min after vinegar rinsing, the proportion of living bacteria stays low, indicating that the proliferation of bacteria was also inhibited, and the effect of vinegar is not transient but long lasting (Fig. 8).

### Saliva

**Fig. 9:**
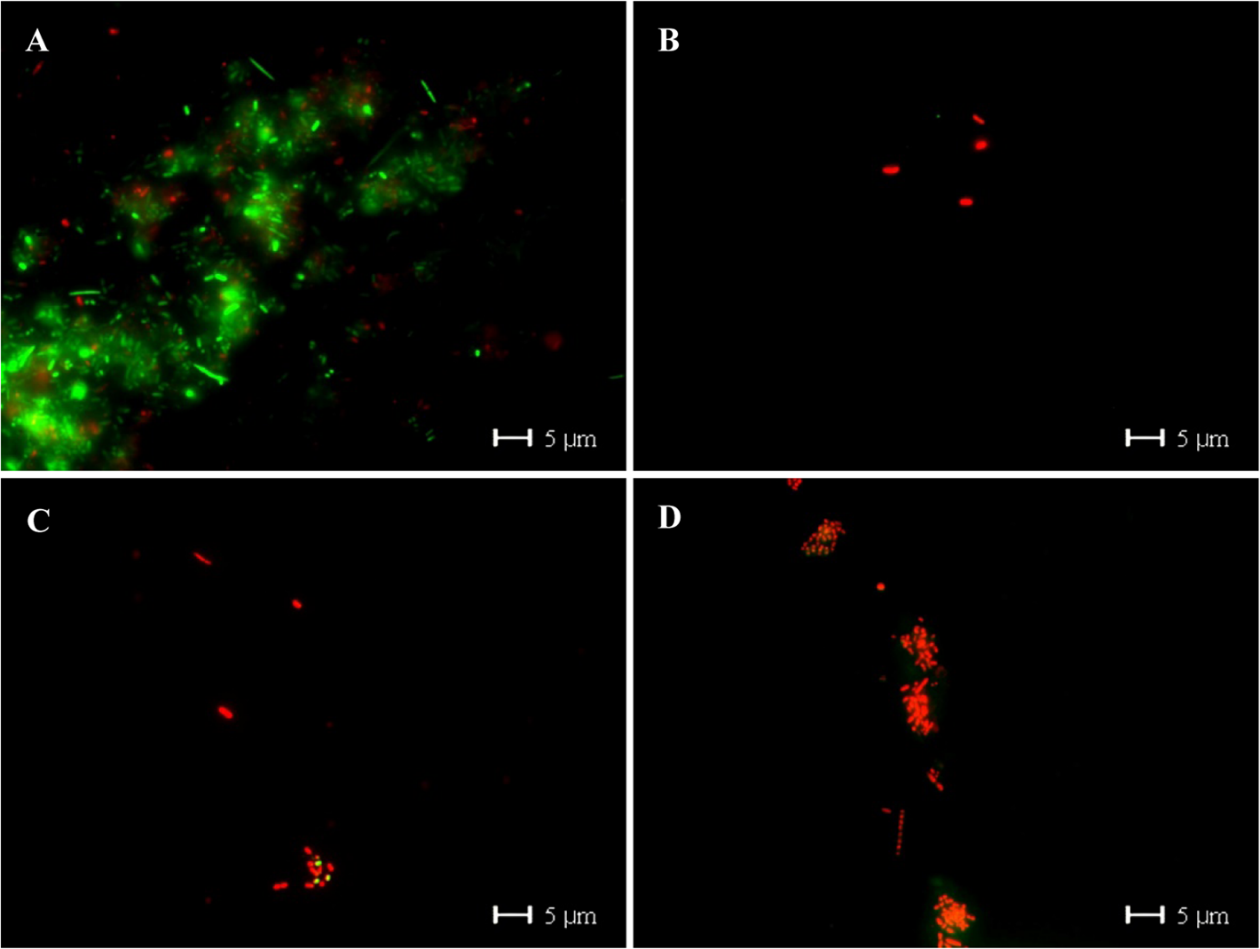
Salivary samples after rinsed with vinegar analyzed by *Bac*Light™ viability assay. The planktonic bacteria in saliva showed significant sensitivities to vinegar within 120 min over time. **a** Control group; **b** immediately after rinsed with vinegar, the salivary bacteria were nearly completely dead; **c** 30 min after rinsed with vinegar; **d** 120 min after rinsed with vinegar. Original magnification: 1000-fold

**Fig. 10:**
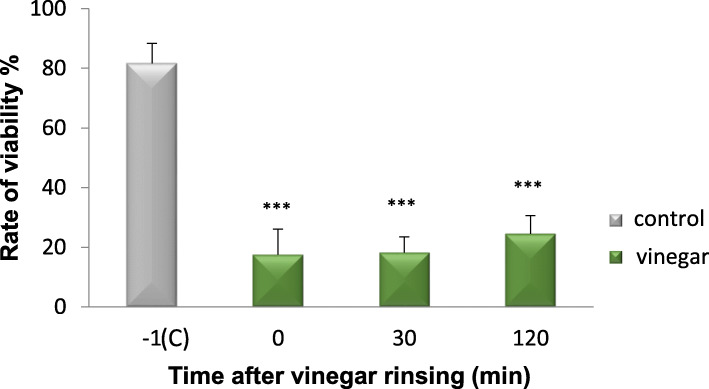
*Bac*Light™ viability assay for determination of bacterial viability in saliva before and after vinegar rinsing at different times. Compared to controls, a significant reduction of bacterial viability was shown after vinegar rinsing for 5 s (*p* < 0.001), after 30 min (*p* < 0.001) and after 120 min (*p* < 0.001). ***p < 0.001

Compared with the embedded bacteria in the 24-h biofilm, vinegar showed stronger lethality to the planktonic bacteria in saliva, which were nearly completely dead. Live bacteria were hardly to be observed immediately after vinegar rinsing and stayed so until 120 min (Fig. 9). The quantification shows the same effect, with bacterial viability decreasing sharply over time (*p* < 0.001) (Fig. 10).

The pH of the vinegar in this experiment was tested to be 2.0, and the pH of the mouthwash of the vinegar was 2.3. 30-min after vinegar rinsing, the oral pH was in the acidic range of 5.5, and after 120 min the pH of the oral cavity was close to the physiological state (pH =6).

## Discussion

The development of biofilms is a dynamic process [2]. Firstly, after contact with saliva and gingival crevicular fluid, the pellicle is formed on the cleansed tooth surface. The main composition of the pellicle are salivary glycoproteins, carbohydrates and lipids which have been examined by analytical techniques [15]. In the 3-min pellicle experiment, the control group presented the normal process of pellicle formation. The thickness of the globular layer increased upon the 120-min experimental trial (*p* < 0.001), which proves former research on pellicle formation [16, 17]. In comparison, these particles layers were removed immediately due to vinegar rinsing, and were sparsely formed during the next 120 min after vinegar rinsing, which suggests that the formation of the pellicle was strongly reduced at least for 120 min. Therefore, vinegar presents substantial inhibition in the initial biofilm formation.

There are few investigations dealing with acetic acid applied on the enamel surface for much longer times than 5 s. These studies revealed dental erosion potential of acetic acid; many of them are in vitro studies [18–21]. According to the daily diet habits, vinegar will not stay in the oral cavity for several minutes due to the swallowing. It has been reported that even the 3-min pellicle could protect the enamel from acid erosion for 60 s [13]. Therefore, short-term application of acid proved to be safe due to the protective effect of the oral biofilm [11]. On the other hand, pure enamel is not exposed intraorally but always covered by biofilms and soaking in saliva in the oral cavity. Thus in situ experiments have advantages in studying erosive processes as compared to in vitro studies [22, 23]. In the 3-min pellicle in situ study, the subsurface pellicle layer below the enamel surface appeared immediately after vinegar rinsing for 5 s with less dense electron appearance as revealed by TEM. This indicated that vinegar could substantially promote the formation of the subsurface pellicle layer, which could prevent further demineralization [11, 15].

After formation of the pellicle, the early bacterial colonizers will adhere to the pellicle surface [2]. In the present experiments, bacteria can already be observed in the control group at 30 min and 120 min after initial 3-min pellicle formation, with many fimbriae firmly fixed on the pellicle surface. Moreover, the quantity of protein particles increased further after the formation of the pellicle for 30 min (*p* < 0.001) as well as the frequency of single microorganisms adhering to the surface in the control group. However, only the enamel surface covered by a thin pellicle with rarely visible protein particles or microbial attachment was observed after vinegar rinsing. Additionally, the vast majority of microorganism in saliva was strongly affected by vinegar rinsing (*p* < 0.001), which results in delay of the early microbial colonization process, thus hindering the formation of biofilm. Therefore, vinegar altered the bacteria in both, initial biofilm and saliva, resulting in the inhibition of early biofilm growth.

After colonized by early microbes, biofilms gradually accumulate a wide variety of other species of bacteria, which promotes growth of the biofilm. It has been reported that bacteria embedded in mature biofilms present more tolerance to antibiotic than planktonic cells as well as antiseptics, biocides and other environmental impacts [24, 25]. Therefore, these biofilm are difficult to remove by antibiotics [26]. In the present study, vinegar presented a stronger antibacterial effect to planktonic bacteria in saliva than bacteria embedded in the 24-h biofilm (*p* < 0.01). Both, TEM as well as SEM images proved that the mature biofilm could functionally protect the microbes better than saliva. Moreover, in the 24-h biofilm experiment, vinegar destroyed the biofilm structure significantly. Especially, 30 min and 120 min after vinegar rinsing, the biofilm was almost wiped out, even the matrix was in part disrupted.

In the oral cavity, saliva as well as the biofilm have buffer capacity for extreme pH values [15, 27, 28]. This buffer capacity is important to maintain the pH value of saliva [29]. In the present experiment, the characteristic of the saliva samples is of great difference immediately after vinegar rinsing (pH = 2.3) compared with the control saliva (pH = 7.0). The control saliva was a very mucus-rich secretion, while the vinegar sample was only a watery fluid essentially devoid of mucus. This suggested that vinegar might change the characteristic of saliva. However, vinegar causes inhibition of biofilm formation as well as biofilm removal, which means that there are some more influencing factors beside the pH value. It has been supposed that salivary flow rate will be increased already before the acid rinsing, which improves the buffer capacity resulting in a protective effect due to the effective dilution of acids [30].

Actually, there are many studies of rinsing solutions used for oral biofilm management and removal, such as chlorhexidine, cetylpyridinium chloride and plant solutions [31–34]. Among them, chlorhexidine is recognized as the gold standard [35]. However, more and more side effects were reported after wide clinical application of chlorhexidine rinsing, such as tooth staining, taste disturbance and even serious allergic reactions [36, 37]. In addition, more recently, concerns have been raised about an enhanced tolerance or even resistance toward CHX that might lead to an enrichment of resistant strains after widespread use of CHX [38–40]. In comparison, vinegar present better advantages in clinical experience. Vinegar cannot avoid bacterial resistance in general, but using formulations containing vinegar instead of antiseptics or antibiotics as oral antibacterial agents may be worthwhile in reducing risk of development of resistance in oral bacteria. As demonstrated in the present study, vinegar reveals a significant efficacy to manage and inhibit short-term biofilm formation. However, further experiments on mature biofilms are needed. These experiments should focus on quantification of the proliferation capacity of bacteria after vinegar treatment, using for example colony-forming unit counts. Furthermore, the effects of vinegar on oral biofilm formation in comparison to gold standard antimicrobials (like CHX) should also be investigated.

## Conclusions

To sum up, this in situ study has demonstrated the potential of the vinegar rinsing approach for inhibition of biofilm formation in the oral cavity, as well as strong removal effect towards the outer globular particles of the initial pellicle on the enamel surface. Although the results of vinegar were significant in this study, the long-term clinical efficacy required further studies.

## Supplementary information

**Additional file 1.**

## Data Availability

The datasets generated and analyzed during the current study are available from the corresponding author on reasonable request. Parts of the present manuscript are content of the first author’s (Yong Liu) thesis [41]. The thesis represents the only medium this content has appeared in, it is in line with the author’s university policy, and can be accessed online. The thesis is present on the university repository website and can be accessed on *https://d-nb.info/1174877146/34*. This article is not published nor is under publication elsewhere.

## Abbreviations

CHX: Chlorhexidine
FM: Fluorescence microscopy
SEM: Scanning electron microscopy
TEM: Transmission electron microscopy

## References

[1] Kassebaum NJ, Smith AGC, Bernabe E (2017). Global, regional, and National Prevalence, incidence, and disability-adjusted life years for Oral conditions for 195 countries, 1990-2015: a systematic analysis for the global burden of diseases, injuries, and risk factors. J Dent Res.

[2] Marsh PD, Lewis, MAO, Williams D, et al. Oral Microbiology. 5th ed. 2009.

[3] Aminifarshidmehr N (1996). The management of chronic suppurative otitis media with acid media solution. Am J Otol.

[4] Jung HH, Cho SD, Yoo CK, Lim HH, Chae SW (2002). Vinegar treatment in the management of granular myringitis. J Laryngol Otol.

[5] Dohar JE (2003). Evolution of management approaches for otitis externa. Pediatr Infect Dis J.

[6] Shay K (2000). Denture hygiene: a review and update. J Contemp Dent Pract.

[7] Bjornsdottir K, Breidt F, McFeeters RF (2006). Protective effects of organic acids on survival of Escherichia coli O157:H7 in acidic environments. Appl Environ Microbiol.

[8] Brul S, Coote P (1999). Preservative agents in foods. Mode of action and microbial resistance mechanisms. Int J Food Microbiol.

[9] De Blackburg C, Mc Clure P. Foodborne Pathogens. 2nd ed. Amsterdam: Elsevier; 2009.

[10] Cherrington CA, Hinton M, Mead GC, Chopra I (1991). Organic acids: chemistry, antibacterial activity and practical applications. Adv Microb Physiol.

[11] Hannig C, Berndt D, Hoth-Hannig W, Hannig M (2009). The effect of acidic beverages on the ultrastructure of the acquired pellicle--an in situ study. Arch Oral Biol.

[12] Hannig C, Hamkens A, Becker K, Attin R, Attin T (2005). Erosive effects of different acids on bovine enamel: release of calcium and phosphate in vitro. Arch Oral Biol.

[13] Hannig M, Fiebiger M, Guntzer M, Dobert A, Zimehl R, Nekrashevych Y (2004). Protective effect of the in situ formed short-term salivary pellicle. Arch Oral Biol.

[14] Deimling D, Hannig C, Hoth-Hannig W, Schmitz P, Schulte-Monting J, Hannig M (2007). Non-destructive visualisation of protective proteins in the in situ pellicle. Clin Oral Investig.

[15] Hannig M, Hannig C (2014). The pellicle and erosion. Monogr Oral Sci.

[16] Hannig M, Balz M (1999). Influence of in vivo formed salivary pellicle on enamel erosion. Caries Res.

[17] Hannig M, Joiner A (2006). The structure, function and properties of the acquired pellicle. Monogr Oral Sci.

[18] da Silva FC, Kimpara ET, Mancini MN, Balducci I, Jorge AO, Koga-Ito CY (2008). Effectiveness of six different disinfectants on removing five microbial species and effects on the topographic characteristics of acrylic resin. J Prosthodont.

[19] de Castro RD, Mota AC, de Oliveira LE, Batista AU, de Araujo OJ, Cavalcanti AL (2015). Use of alcohol vinegar in the inhibition of Candida spp. and its effect on the physical properties of acrylic resins. BMC Oral Health.

[20] Meurman JH, ten Cate JM (1996). Pathogenesis and modifying factors of dental erosion. Eur J Oral Sci.

[21] Willershausen I, Weyer V, Schulte D, Lampe F, Buhre S, Willershausen B (2014). In vitro study on dental erosion caused by different vinegar varieties using an electron microprobe. Clin Lab.

[22] Wake N, Asahi Y, Noiri Y (2016). Temporal dynamics of bacterial microbiota in the human oral cavity determined using an in situ model of dental biofilms. Biofilms Microbiomes.

[23] Wiegand A, Bliggenstorfer S, Magalhaes AC, Sener B, Attin T (2008). Impact of the in situ formed salivary pellicle on enamel and dentine erosion induced by different acids. Acta Odontol Scand.

[24] Blanc V, Isabal S, Sanchez MC (2014). Characterization and application of a flow system for in vitro multispecies oral biofilm formation. J Periodontal Res.

[25] Charlebois A, Jacques M, Boulianne M, Archambault M (2017). Tolerance of Clostridium perfringens biofilms to disinfectants commonly used in the food industry. Food Microbiol.

[26] Hwang G, Koltisko B, Jin X, Koo H (2017). Nonleachable Imidazolium-incorporated composite for disruption of bacterial clustering, exopolysaccharide-matrix assembly, and enhanced biofilm removal. ACS Appl Mater Interfaces.

[27] Garcia-Godoy F, Hicks MJ (2008). Maintaining the integrity of the enamel surface: the role of dental biofilm, saliva and preventive agents in enamel demineralization and remineralization. J Am Dent Assoc.

[28] Martins C, Castro GF, Siqueira MF, Xiao Y, Yamaguti PM, Siqueira WL (2013). Effect of dialyzed saliva on human enamel demineralization. Caries Res.

[29] Bardow A, Moe D, Nyvad B, Nauntofte B (2000). The buffer capacity and buffer systems of human whole saliva measured without loss of CO2. Arch Oral Biol.

[30] Hara AT, Zero DT (2014). The potential of saliva in protecting against dental erosion. Monogr Oral Sci.

[31] Dabholkar CS, Shah M, Kathariya R, Bajaj M, Doshi Y (2016). Comparative evaluation of antimicrobial activity of pomegranate-containing mouthwash against Oral-biofilm forming organisms: an Invitro microbial study. J Clin Diagn Res.

[32] Pitten FA, Kramer A (2001). Efficacy of cetylpyridinium chloride used as oropharyngeal antiseptic. Arzneimittelforschung.

[33] Santos GOD, Milanesi FC, Greggianin BF, Fernandes MI, Oppermann RV, Weidlich P (2017). Chlorhexidine with or without alcohol against biofilm formation: efficacy, adverse events and taste preference. Braz Oral Res.

[34] Sreenivasan PK, Haraszthy VI, Zambon JJ (2013). Antimicrobial efficacy of 0.05% cetylpyridinium chloride mouthrinses. Lett Appl Microbiol.

[35] Jones CG (1997). Chlorhexidine: is it still the gold standard?. Periodontol.

[36] Bahal S, Sharma S, Garvey LH, Nagendran V (2017). Anaphylaxis after disinfection with 2% chlorhexidine wand applicator. BMJ Case Rep.

[37] Flotra L (1973). Different modes of chlorhexidine application and related local side effects. J Periodontal Res Suppl.

[38] Cieplik F, Jakubovics NS, Buchalla W, Maisch T, Hellwig E, Al-Ahmad A (2019). Resistance toward chlorhexidine in oral bacteria - is there cause for concern?. Front Microbiol.

[39] Wand ME, Bock LJ, Bonney LC, Sutton JM (2016). Mechanisms of increased resistance to Chlorhexidine and cross-resistance to Colistin following exposure of Klebsiella pneumoniae clinical isolates to Chlorhexidine. Antimicrob Agents Chemother.

[40] Horner C, Mawer D, Wilcox M (2012). Reduced susceptibility to chlorhexidine in staphylococci: is it increasing and does it matter?. J Antimicrob Chemother.

[41] Yong L (2017). Influence of vinegar on biofilm formation in situ; Thesis.

