# Supplementary material for: Vinegar inhibits the formation of oral biofilm in situ
Source: BMC Oral Health. 2020 Jun 5;20:167. doi: 10.1186/s12903-020-01153-z (PMC7275295; doi:10.1186/s12903-020-01153-z)
Supplement: Supplementary file 1 — Additional file 1. [file 12903_2020_1153_MOESM1_ESM.pptx]

## Slide 1
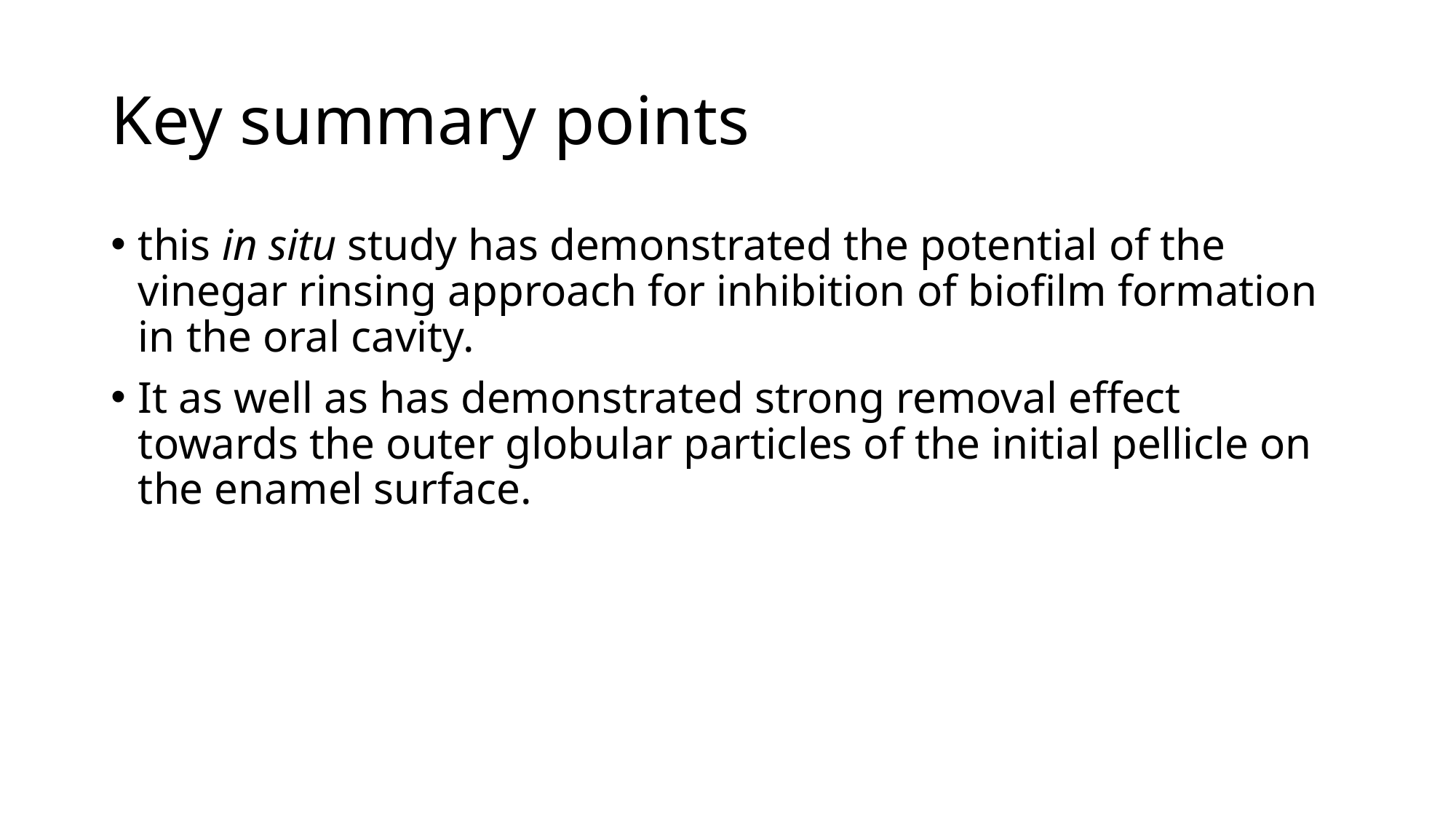

# Key summary points
this in situ study has demonstrated the potential of the vinegar rinsing approach for inhibition of biofilm formation in the oral cavity.
It as well as has demonstrated strong removal effect towards the outer globular particles of the initial pellicle on the enamel surface.
